# Open letter to journal editors on: International Consensus Radiochemistry Nomenclature Guidelines

**DOI:** 10.1186/s41181-018-0047-y

**Published:** 2019-04-16

**Authors:** Heinz H. Coenen, Antony D. Gee, Michael Adam, Gunnar Antoni, Cathy S. Cutler, Yasuhisa Fujibayashi, Jae Min Jeong, Robert H. Mach, Thomas L. Mindt, Victor W. Pike, Albert D. Windhorst

**Affiliations:** 1 Research Centre Jülich, Jülich, Germany; 2 King’s College London, London, UK; 3 TRIUMF, Vancouver, Canada; 4 Uppsala University, Uppsala, Sweden; 5 Brookhaven National Laboratory, Upton, USA; 6 Keio University, Tokyo, Japan; 7 Seoul National University, Seoul, South Korea; 8 University of Pennsylvania, Philadelphia, USA; 9 Ludwig Boltzmann Institute of Applied Diagnostics, Vienna, Austria; 10 National Institute of Mental Health, Bethesda, USA; 11 VU University Medical Center, Amsterdam, The Netherlands

Dear Editors,

After observing an increased incidence of imprecise and sometimes erroneous use of nuclear chemistry, radiochemistry and radiopharmacy related terms and nomenclature in scientific reports, an international working group of experts was assembled to address the issue. Upon extensive consultation with peers within the field of radiochemistry and radiopharmaceutical sciences over a 3-year period and an open discussion, consensus was achieved during the International Symposium on Radiopharmaceutical Sciences in May 2017 in an open forum. The resulting, harmonised nomenclature recommendations have now been published with following aims given therein (Coenen et al. 2017):

- Provide a reference source for nomenclature good practice in the radiopharmaceutical sciences.
- Clarify the use of terms and rules concerning exclusively radiopharmaceutical terminology, i.e. nuclear- and radiochemical terms, symbols and expressions.
- Address gaps and inconsistencies in existing radiochemistry nomenclature rules.
- Provide source literature for further harmonisation beyond our immediate peer group (publishers, editors, IUPAC, pharmacopoeias, etc.).

In order to disseminate further the consensus recommendations, a summary (see below) has been prepared for ease of reference and for open dissemination across the field. We hope that your journal will consider incorporating these guidelines as part of your journals manuscript preparation instructions.

Yours sincerely,

## International consensus nomenclature guidelines – Summary

### Measures of radioactivity

#### Unit of activity

“***Radioactivity***” is a physical *phenomenon*, defined as the *property* of certain nuclei to spontaneously fragment or rearrange, resulting in the emission of radiation.

“***Activity***” is the *quantitative measure* of radioactivity: The number of nuclear decays, occurring in a given quantity of material over a certain time interval, divided by that time interval.

“***Becquerel***” **(Bq)** is the agreed SI derived unit for the *quantity* of activity.

Pre-SI units (e.g. imperial units) (e.g. mCi, Ci) can also be used, but must be placed in parentheses after the stated SI units.

N.B.: The correct terms “radioactive” and “non-radioactive” must not be replaced by the lab-jargon “hot” and “cold”, respectively, in public or official documents.

#### Molar activity (A_m_) and specific activity (A_s_)

***"Molar activity"*** is the measured activity **per mole** of compound; measured in Bq/mol (GBq/μmol).

***"Specific activity"*** is the measured activity **per gram** of compound; measured in Bq/g (GBq/μg).

Due to radioactive decay, the instant of time of measurement must be stated; for example: “The molar activity was 50 GBq/μmol 2 h after the end of synthesis”.

#### Apparent molar activity and apparent specific activity

The terms "apparent molar activity" and "apparent specific activity" take into account the amounts of the labelled and non-radiolabelled impurities present (using moles, or weight, respectively).

#### Effective molar activity and effective specific activity

The terms “effective molar activity” and “effective specific activity” address the chemically, biologically or pharmacologically ‘active’ fraction of radioactive and non-radioactive materials in a sample, competing with the labelled product in its chemical or biological reactions.

In this case, the “effectivity” must be determined by an additional analytical process; e.g. receptor or enzyme binding assay, side-product analysis, etc.

Other terms like “pseudo-specific activity” etc. must not be used.

#### ‘No-carrier-added’, ‘carrier added’ and ‘carrier-free’

The non-quantitative terms, “no-carrier-added” (n.c.a.) and “carrier-added” (c.a.) can be used as a practical, qualitative indication of specific or molar activity.

The term “carrier-free” (c.f.) can only be used in the rare case where the theoretical maximum specific or molar activity is unambiguously proven.

Measures involving determination of amounts of material (e.g. molar activity or radiochemical purity) should be accompanied by a clear description of the method of detection.

### Radionuclide and radioisotope descriptors

The enrichment of a chemical compound with an isotope (stable or radioactive) of one or more of the elements, of which it is constituted, is indicated by the symbol of the element (E) together with its mass number (A) (as a superscript in front) within square brackets, [^A^E], immediately preceding the compound’s name or chemical formula.

Example: [^2^H,^14^C] benzene, or [^2^H,^14^C]C_6_H_6_, represent the compound *benzene*, enriched or labelled, with stable deuterium and radioactive carbon-14, respectively.

For rules designating labelling positions (e.g. L-[*methyl*-^11^C] methionine or L-[*carboxyl*-^11^C]methionine) see: http://goldbook.iupac.org/pages/about.html*.*

These rules apply equally to organic, inorganic and organometallic compounds and complexes: e.g. [^223^Ra]RaCl_2_, [^99m^Tc]NaTcO_4_, [^99m^Tc]Tc-MDP, [^68^Ga]Ga-DOTA-TATE, etc.

As square brackets are also used to denote metal complexes, care should be taken to avoid confusion with radionuclide descriptors, e.g. [^99m^Tc][Tc (CO)_3_(OH_2_)_3_]^+^, [^111^In][In (DTPA)]^2−^, etc.

N.B.: The symbol for isotopic enrichment [^A^E] should be treated like a syllable, and thus only be hyphenated at the end of a line of text.

Conversely, do not use nuclide symbols in square brackets in combination with nouns and verbs.

Examples of the use of square brackets and hyphens:

**Table:** 

Correct	Incorrect
L-[^13^N] alanine, (S)-[^13^N]alanine	[^13^N]L-alanine, (S)-^13^N-alanine, L-[^13^N]-alanine
[^18^F]fluorobenzene	[^18^F]benzene (no fluorine atom in benzene)
[^99m^Tc]TcDTPA^2−^	[^99m^Tc]DTPA^2−^, ^99m^Tc-DTPA^2−^ (no Tc in chelator)
^11^C-compound, ^125^I-substitution, ^18^F-derivative, ^68^Ga-conjugate, ^77^Br-reagent	[^11^C]compound, [^125^I]-substitution, [^18^F]-derivative, [^68^Ga]conjugate (no chemical compound names)
^11^C-labelling, ^64^Cu-labelling, ^18^F-fluorination (with hyphen!)	[^11^C]labelling, [^64^Cu]-labelling, [^18^F]-(radio)-fluorination (no chemical compound names)

The terms ‘(radio)isotope’ and ‘(radio)nuclide’ are often used incorrectly in texts, inferring “isotope” means “radioactive nuclide” or even “labelled compound”.

- ‘Nuclide’ indicates an atom, characterised by its numbers of protons (identifying its elemental nature) and of nucleons (indicating its mass).
- ‘Isotopes’ are nuclides of the same element (same proton number), but having different numbers of neutrons (hence different atomic mass).
- Isotopic nuclides of different energy state are called isomeric nuclides, isomeric isotopes or ‘isomers’, such as technetium-99g and -99m.

N.B.: All (radio)isotopes are (radio)nuclides, while the reverse is not true!

### Radiochemical yield (RCY)

The “radiochemical yield” is the amount of activity in the product expressed as the percentage (%) of related starting activity utilized in the considered process (e.g. synthesis, separation, etc.). The quantity of both must relate to the same radionuclide and be decay corrected to the same point in time before the calculation is made.

(Reported measures of RCY should indicate, whether a product was isolated or not.)

Colloquial expressions for “radio-yield” found in literature, e.g. ‘radiochemical conversion’, ‘analytical radiochemical yield’, ‘radio-HPLC yield’ must not be used as a surrogate for ‘radiochemical yield’ or ‘radiochemical purity’, respectively.

The following text may serve as examples for good practice, when describing a radiochemical yield:

“The radiochemical yield was 67% (based on HPLC analysis of the crude product).”

“The radiochemical yield* of “Y” was 67%”, with the following as a footnote:

*“determined by radio-HPLC analysis of the crude product”, or
*“non-isolated, estimated by radio-HPLC”,

or, in the general experimental section: “All radiochemical yields were determined by HPLC of the crude product, unless stated otherwise.”,

or alternatively use: “The radiochemical purity of the crude product was 67%.”,

or: “The radiochemical yield of “Y” determined from an aliquot of the reaction solution amounted to 67%.”,

or: “The radiochemical yield of crude “Y” was 67% based on the amount of activity eluted from the HPLC column.”

Expressions such as ‘conversion’ or ‘incorporation’, however, may be used in a semantic sense and even be indispensable in context of mechanistic discussions. For example, “The ‘conversion’ (or ‘incorporation’) proceeded with a 50% yield”. Here it is clear from the context, that the radiochemical yield of the conversion is intended.

#### Activity yield

The “activity yield” is the amount of radioactive product expressed in Bq (MBq, GBq), which is obtained from a starting amount of activity (e.g. produced at a cyclotron) and is not corrected for decay.

This term is useful, or necessary to indicate the efficiency of a labelling procedure. The activity yield is, of course, dependent on the effectiveness and duration of all technical manipulations used, in addition to the yield of the labelling reaction.
